# The final preamble

**DOI:** 10.1017/S1478951526102338

**Published:** 2026-04-20

**Authors:** Zhaohui Su

**Affiliations:** School of Public Health, Southeast University, Nanjing, China

Enough preamble, you say.

As if death by seppuku,
The goodbye has to be said.

Struggled over every word,
Pined over every line ahead.
Perhaps still hiding from the truth,
Perhaps choosing not to lie instead.

Enough preamble, you say.

As if a study in how death imitates art,
The goodbye has to be shared.

How does one get from matter to mind?
How is a soul’s ending prepared?
Perhaps a goodbye is just a goodbye,
A final breath of empty air.

Enough preamble, you say.

As if reminiscing a book of love from afar.
The goodbye has to be declared.

As there is joy in the First-Time-Human Tribe,
There is peace in the Last-Life Club.
Perhaps silver linings are the default,
Perhaps all of life ought to be loved.

Enough preamble, you say.

As if desired but not claimed,
The goodbye has to be aired.

What we had was fun,
A smile-then-sigh kind of affair.
In memory, everything stays prime,
Perhaps that’s where we shall leave everything at.

## Data Availability

Data are available upon reasonable request.

